# Supplementary material for: Complexome profiling of the Chlamydomonas psb28 mutant reveals TEF5 as an early PSII assembly factor
Source: Plant Cell. 2025 Mar 17;37(6):koaf055. doi: 10.1093/plcell/koaf055 (PMC12164590; doi:10.1093/plcell/koaf055)
Supplement: koaf055_Supplementary_Data [file koaf055_supplementary_data.zip › psb28_interactive.zip!/plots/intensity/qualityPlots/peptides/ACS3_Cre07.g353450.t1.2.html]

### Cre07.g353450.t1.2

Trivial name: ACS3  
SeqCoverage [median]: 31.30  
Mass: 73.22 kDa  
  
 

| Sequence | MissedCleavage | Mass [Da] | wtIntensity | muIntensity | Score |
| --- | --- | --- | --- | --- | --- |
| ELLAEVNR | 0 | 942.51 | 7764.34 | 9949.32 | 117.93 |
| FESAYFAPYK | 0 | 1221.57 | 1268.64 | 6258.28 | 99.14 |
| GEQAALIWEGNDENERR | 1 | 1985.92 | 470.83 | 1654.03 | 54.86 |
| GHMAANTDIR | 0 | 1084.51 | 3424.96 | 2653.71 | 99.81 |
| GQAIYAFVTLR | 0 | 1237.68 | 859.35 | 0.00 | 71.35 |
| GTKPVLLK | 1 | 854.56 | 0.00 | 628.65 | 86.79 |
| GVWPGMMR | 0 | 932.44 | 0.00 | 287.39 | 56.51 |
| IASGEEKELGDISTLAEPGVVDMLIK | 1 | 2713.41 | 854.17 | 0.00 | 43.10 |
| NDYDTMYKR | 1 | 1204.52 | 1984.44 | 727.54 | 55.57 |
| QFYTAPTLLR | 0 | 1208.66 | 3683.82 | 3439.16 | 89.25 |
| QSDLSSLR | 0 | 904.46 | 6072.76 | 1134.29 | 95.53 |
| SFLLYTSGSTGKPK | 1 | 1484.79 | 15304.22 | 32760.30 | 88.44 |
| SLLQLGDAWPR | 0 | 1254.67 | 1394.21 | 0.00 | 92.06 |
| TIADDAVK | 0 | 831.43 | 3529.60 | 2529.56 | 73.26 |
| TIADDAVKLAAK | 1 | 1214.69 | 552.40 | 2905.84 | 72.29 |
| TPLLATK | 0 | 742.46 | 5539.16 | 0.00 | 71.30 |
| VGAVHSVVFAGFSADSLAQR | 0 | 2017.04 | 10629.23 | 16075.86 | 84.03 |
| VLLTATGVMR | 0 | 1059.61 | 41010.24 | 33157.63 | 163.15 |
|  |  |  | 104342.4 | 114161.6 |  |
